# Emerging Roles of microRNAs as Biomarkers and Therapeutic Targets for Diabetic Neuropathy

**DOI:** 10.3389/fneur.2020.558758

**Published:** 2020-10-22

**Authors:** Baoyan Fan, Michael Chopp, Zheng Gang Zhang, Xian Shuang Liu

**Affiliations:** ^1^Department of Neurology, Henry Ford Health System, Detroit, MI, United States; ^2^Department of Physics, Oakland University, Rochester, MI, United States

**Keywords:** diabetic neuropathy, microRNA, neurovascular dysfunction, extracellular vesicle (EV), exosome

## Abstract

Diabetic neuropathy (DN) is the most prevalent chronic complication of diabetes mellitus. The exact pathophysiological mechanisms of DN are unclear; however, communication network dysfunction among axons, Schwann cells, and the microvascular endothelium likely play an important role in the development of DN. Mounting evidence suggests that microRNAs (miRNAs) act as messengers that facilitate intercellular communication and may contribute to the pathogenesis of DN. Deregulation of miRNAs is among the initial molecular alterations observed in diabetics. As such, miRNAs hold promise as biomarkers and therapeutic targets. In preclinical studies, miRNA-based treatment of DN has shown evidence of therapeutic potential. But this therapy has been hampered by miRNA instability, targeting specificity, and potential toxicities. Recent findings reveal that when packaged within extracellular vesicles, miRNAs are resistant to degradation, and their delivery efficiency and therapeutic potential is markedly enhanced. Here, we review the latest research progress on the roles of miRNAs as biomarkers and as potential clinical therapeutic targets in DN. We also discuss the promise of exosomal miRNAs as therapeutics and provide recommendations for future research on miRNA-based medicine.

## Introduction

Diabetic neuropathy (DN) is a prevalent complication associated with diabetes mellitus (1). Approximately half of all diabetic patients, either pre-diabetes, type 1 or type 2 diabetes, will go on to develop this condition (2, 3). The symptoms of DN vary according to different stages of the disease. However, the commonality across all stages is the distal-to-proximal gradient of severity (4, 5). DN involves more sensory than motor impairments (6, 7). At the early stage, DN patients mainly experience pain and hyperalgesia. As the disease progresses, patients experience numbness, muscle weakness, loss of balance, and foot ulcers (6, 8, 9). Besides the staggering healthcare costs, DN patients experience poor quality of life and have high rates of ulcerations and amputations (10, 11). Also, long-term physical discomfort may lead to the development of depression and anxiety (12).

To date, the treatment options for DN patients include (1) intensive glycemic control, which slows the progression of the disease, (2) pain relief drugs, anti-seizure medications, and antidepressants, and (3) foot care (13, 14). However, a recent meta-analysis of DN studies has indicated that glycemic control does not benefit the majority of DN patients (15). Additionally, rapid glucose lowering can trigger neuropathic pain, which is known as treatment-induced neuropathy (15). Thus, available treatments for DN are far from sufficient.

## Function of microRNAs

Increasing evidence indicates that microRNAs (miRNAs) are involved in the pathogenesis of DN (16). miRNAs are non-coding RNA sequences, and they are composed of 18–24 nucleotides in length (17). miRNAs bind to target mRNAs and induce translational repression and mRNA decay (18). Mature miRNAs can be released into the circulation and body fluids. As they are protected by RNA-binding proteins and lipid-containing vesicles (microvesicles, exosomes, apoptotic bodies, and high-density lipoprotein), miRNAs show good stability and facilitate communication between cells or organs (19). In the context of diabetes, hyperglycemia, hypoxia, and inflammation affect miRNA biogenesis and release. Consequently, these alterations in the miRNA profile are associated with multiple microvascular complications (20–23). Of note, a significant portion of miRNAs was found to be specifically packaged into extracellular vesicles that express cell-type-specific proteins to mediate their different functions (24). These findings support the potential applications of miRNAs in the diagnosis and therapy for DN (25).

In this review, we summarize recent findings on the roles of miRNAs in the progression of DN and their potential as biomarkers and therapeutic targets of DN. In particular, we emphasize the clinical potential of miRNA-based therapy in the treatment of DN.

## miRNAs and the Pathophysiology of Diabetic Neuropathy

**Figure 1:**
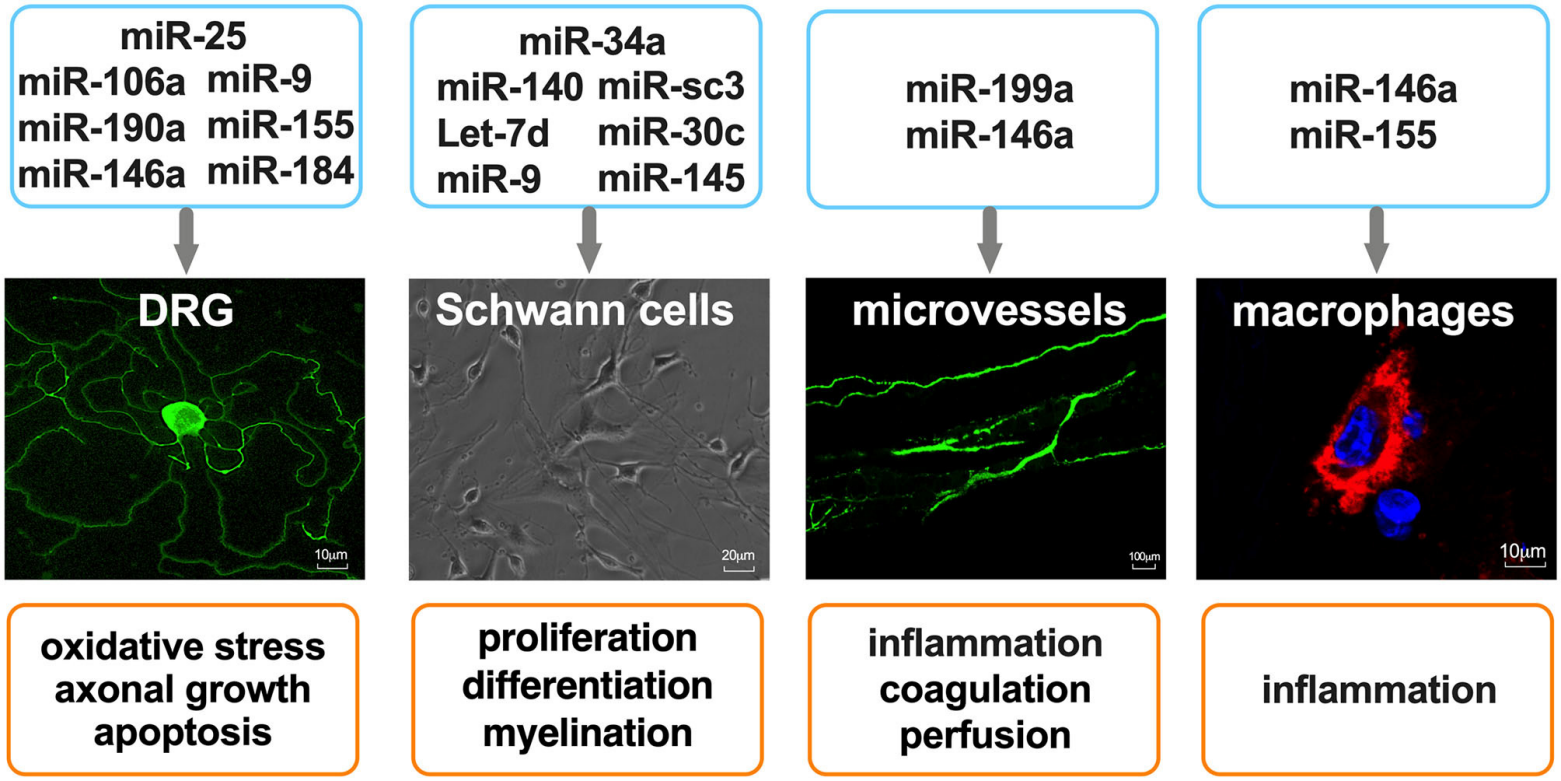
miRNAs as regulators of cell function in the peripheral nervous system. Primary mouse DRG neurons were stained with anti-Neurofilament H. Microvessels in mouse sciatic nerves were perfused with FITC-dextran (molecular mass 2,000 kDa, 500 mg/kg). Macrophages were stained with CD68, and nuclei were stained with 4′,6-diamidino-2-phenylindole.

DN is a multifunctional disorder which is characterized by complex pathogenic mechanisms that have not been fully elucidated. The pathogenic factors which contribute to DN include metabolic dysfunction, inflammation, and oxidative stress, the associated molecular underpinnings of which have been proposed (26, 27). In this subsection, we discuss recent findings regarding the roles that miRNAs play in the regulation of peripheral nervous system (PNS) function and their therapeutic potential in DN (Figure 1).

### miRNAs and Metabolic Alteration of Diabetic Neuropathy

The metabolic changes in diabetes that contribute to DN include hyperglycemia, dyslipidemia, and insulin resistance (28–33). These metabolic imbalances promote the activation of protein kinase C (PKC), polyol and hexosamine pathways, advanced glycation end products (AGEs) production, the activity of poly (ADP-ribose) polymerase (PARP), lipoxygenase, and elevated Na^+^/H^+^ exchanger-1, as well as suppression of insulin signaling (34–43). Oxidative stress and inflammation are considered as critical common pathways of cellular injury in hyperglycemia (44–46). An increase in oxidative and nitrosative stress in plasma and tissues cause mitochondrial impairment, DNA damage and apoptosis (47–49). Studies have reported the role of miRNAs in oxidative response in diabetes but reports on DN individuals are limited. In db/db mice with DN, overexpression of the miR-25 precursor reduced PKC-α phosphorylation-mediated reactive oxygen species production in diabetic peripheral nerves, leading to improvement of neurological function (50). Wu et al. reported that miR-106a targeted 12/15-Lipoxygenase of oxidative/nitrative stress and hindered apoptosis of dorsal root ganglia (DRG) in DN (51). However, the important role of miRNAs in the regulation of metabolism associated with DN remains to be determined.

### miRNAs and Inflammation of Diabetic Neuropathy

DN is closely associated with chronic low-grade inflammation and activation of the innate immune system (27). Inflammation is strongly associated with neuropathic pain, which affects approximately 10–40% of the general population (52). In DN patients, elevated glucose levels increase fibronectin and inflammatory mediators, such as IL-6, vascular endothelial growth factor (VEGF), nuclear factor-kappa B (NF-κB), TNFα, intercellular adhesion molecule-1 (ICAM-1), and transforming growth factor-β (TGF-β). These effects cause an increased sensitization of the central nervous system (53–57). Gong et al. examined the miRNA profiles of the lumbar spinal dorsal horn of mice with DN and found that miR-190a-5p and miR-184-5p were related to inflammation-associated genes that were involved in the pathogenesis of neuropathic pain (58). MiR-9 interacted with calcium homeostasis modulator 1 (CALHM1) and activated the ATP-P2X7R signaling pathway in the spinal cord neurons of rats with DN (59). MiR-155 mediated neuropathic pain by targeting NF-κB activity (60).

Using rodent models, some miRNA-based treatments have been shown to be effective in pain control (Table 1). MiR-190a alleviated pain by targeting solute carrier family 17 member 6 (SLC17A6) and decreasing the IL-1β and IL-6 level in the lumbar spinal dorsal horn (61). In a mouse model of DN, miR-155 mimics suppressed pro-inflammatory cytokines and attenuated DN by targeting tumor necrosis factor receptor-associated factor 2 (TRAF2) and Notch2 (62). Zhang et al., reported that miR-25 decreased reactive oxygen species content in diabetic peripheral nerves by activating NADPH oxidase and upregulating Advanced glycation endproduct (AGE)–RAGE interaction (50), In a diabetic state, reduced miR-146a expression was observed in serum, leukocytes, retina, heart, and PNS (65–67). MiR-146a was also reported to be involved in DN progression (64, 68). Our recent experimental study adds substantially to this body of evidence (63). The administration of miR-146a mimics increased nerve conduction velocity and ameliorated morphological damage in the sciatic nerve and footpad of DN mice. In the circulation and the PNS, miR-146a reduced levels of the inflammatory cytokines TNFα and IL-1β. Further analysis revealed that miR-146a altered the macrophage polarization by suppressing proinflammatory macrophage (M1) activation and increasing anti-inflammatory macrophage (M2) activation (63). These results are consistent with those of other groups, which emphasize the regulatory effects of miR-146a on inflammatory responses in DN (64, 68–70).

**Table 1:** miRNAs and the regulation of inflammation in DN model.

**miRNAs**	**Chromosomal location**	**Target genes/regulated pathways**	**DN model**	**References**
miR-190a-5p	chr9 (mouse)	SLC17A6	Balb/c mice injected with STZ (200 mg/kg)	(61)
miR-155	chr11 (rat)	NF-κB	SD rats* injected with STZ (50 mg/kg)	(60)
	chr16 (mouse)	TRAF2, Notch2	db/db mice	(62)
miR-9	chr2 (rat)	CALHM1	SD rats injected with STZ (60 mg/kg)	(59)
miR-25	chr5 (mouse)	AGE–RAGE pathway	db/db mice	(50)
miR-146a	chr11 (mouse)	IRAK1, TRAF6	db/db mice	(63, 64)

* *SD rats, Sprague-Dawley rats*.

### Roles of miRNAs in the Neurovascular Dysfunction of Diabetic Neuropathy

#### miRNAs and Sensory Neurons

Approximately 80% of DN patients develop gradual damage to the distal terminals of sensory neurons, leading to the symptoms of pain or loss of sensation in their toes (4). Diabetes preferentially targets sensory nerves, autonomic nerves and then motor nerves (3). An explanation is that sensory neurons located in the DRG lack the protection of the blood-nerve barrier, while motor neurons located in the ventral horn of spinal cord retain this protection (71). Furthermore, the PNS contains more unmyelinated axons, known as C fibers than myelinated axons. C fibers send afferent impulses in response to thermal and mechanical stimuli (72). Loss of C fibers is an early change that is witness to the development of DN (73). The molecular mechanisms of how diabetes targets peripheral neurons and their axons remain unclear. The prevailing hypotheses include oxidative stress, AGEs accumulation, inflammation, and mitochondrial dysfunction, all of which contribute to the injury of DRG neurons and alteration of mRNA and miRNA expression (74–76). Cheng et al. found that Let-7i and miR-341 were dysregulated in diabetic DRG, while administration of let-7i mimic and anti-miR-341 independently improved structural abnormalities and neurological dysfunction in a mouse model of DN (75). MiR-29b was found to be down-regulated in DRG neurons of STZ-induced diabetic rats. The decreased miR-29b was associated with axonal swelling, apoptosis, and abnormal gene profiles in DRG (77).

In order to reverse the neuropathic deficits associated with DN, another strategy involves the activation of axonal regeneration, which has shown promising results in *in vitro* and *in vivo* DN models (78–80). PNS neurons are capable of long-distance axonal regeneration (81). The administration of miR-29b mimic promoted axonogeneration and inhibited neurodegeneration via the TGF-β/Smad3 pathway (77). Previous studies from our group showed that miRNAs mediate the axonal growth of DRG neurons. Diabetic db/db mice showed upregulation of miR-29c in peripheral nervous tissues. When cultured under high glucose condition, DRG neurons showed a substantial reduction in axonal growth. However, when the DRG neurons were transfected with a miR-29c inhibitor, attenuation of endogenous miR-29c increased axonal growth. This effect was mediated by PRKCI, which codes for Protein kinase C iota. When PRKCI was knocked down, the effects of miR-29c mimics or inhibitors were abolished (80).

In another study, our group observed that miR-34a was involved in axonal growth in db/db mice with DN. In cultured DRG neurons, miR-34a targets a seed region in the 3′UTR region of forkhead box protein P2 and vesicle amine transport 1, leading to a reduction in axonal growth (79). Hyperglycemia down-regulates miR-146a, which is involved in the reduction of axonal outgrowth and apoptosis of DRG neurons (78, 82). Axonal miRNAs locally regulate energy metabolism. Using DRG neurons cultured in a microfluidic chamber, high glucose locally reduced miR-146a levels in distal axons. Gain- and loss-of-function of miR-146a regulate axonal growth via its target genes IL-1 receptor-associated kinase 1(IRAK1) and tumor necrosis factor receptor-associated factor 6 (TRAF6) (78).

#### miRNAs and Schwann Cells

Schwann cells are the most abundant glial cells in the PNS, and they maintain neuronal structure and function, promote survival, and growth after injury (83, 84). Sciatic nerves of DN patients usually show Schwann cell injury. The ultrastructure of Schwann cells shows reactive, degenerative, and proliferative changes with the progression of DN (83).

In a diabetic state, hyperglycemia interrupts Schwann cell function through different mechanisms (85). Redundant glucose is converted to sorbitol by aldose reductase, which is expressed in myelinating Schwann cells, and depletes NADPH, leading to oxidative stress (86). In addition, sorbitol promotes Schwann cell dedifferentiation into an immature phenotype via reducing insulin-like growth factor 1 expression (87). Excess glucose and modified lipoproteins trigger the production of inflammatory cytokines in Schwann cells and thereby enhance immune cell recruitment (88). Diabetes-induced mitochondria dysfunction activate a maladaptive stress response in Schwann cells, which induces lipid oxidation and inhibits lipid synthesis, resulting in the depletion of the lipid myelin components, and thereby causes axonal degeneration (89).

Schwann cells can sense axonal loss and dedifferentiate to a proliferative phase and support axonal regeneration (83). The immature-like Schwann cells clear myelin debris and release neurotrophic factors until new axons form. Then, they redifferentiate to form myelin sheaths (84). Using *in vitro* and *in vivo* DN models, miRNAs were demonstrated to participate in the response of Schwann cells to injury and Schwann cell–axon interactions (90). A recent study showed that Dicer, which is a critical enzyme for miRNA biogenesis, is important for Schwann cell myelination (91). Viader et al. found that miR-34a was able to regulate dedifferentiation by Notch1 and cyclin D1 (92). MiR-140 was found to regulate the expression of the transcription factor for myelinogenesis, Krox20 (92). Furthermore, Let-7d significantly reduced primary Schwann cell proliferation and migration by directly targeting nerve growth factors (93). MiR-9, miR-sc3, miR-30c were reported to be involved in Schwann cell proliferation and migration (94–96). In cultured rat Schwann cells (RSC96), the inhibition of miR-145-3p by circular RNA ACR alleviated high glucose-induced cell apoptosis, autophagy, and ROS generation by activating the phosphatidylinositol 3-kinase/protein kinase B/mammalian target of rapamycin (PI3K/AKT/mTOR) pathway (97). Therefore, manipulation of Schwann cell miRNAs has the potential to be used therapeutically to reverse the effects of DN.

#### miRNAs and Microvascular Dysfunction

DN is considered to be a microvascular complication of diabetes (98). Several mechanisms of hyperglycemia-induced cellular injury were described to occur in the vascular endothelium (99, 100). Nerve biopsies from DN patients show thickening of capillary basement membrane and capillary pericyte degeneration (98). Microvasculature dysfunction results in a reduction of nutrition, oxygen, and waste transportation through nerves, and either precedes or parallels nerve dysfunction and demyelination (98, 101). Although the mechanism is not fully understood, there is evidence that the chronic inflammatory state of diabetes contributes to microvascular complications (102).

miRNAs are involved in diabetes-induced vascular dysfunction (103). In a study of 60 patients with DN, miR-199a-3p was significantly higher compared to control volunteers. The authors proposed that miR-199a-3p promotes coagulation of the skin peripheral circulation through down-regulating serine protease inhibitor E2 (SerpinE2) and up-regulating matrix metalloproteinase-13 (MMP-13) in endothelial cells (104). Kamali et al. reported that miR-146a is upregulated in human umbilical vein endothelial cells during the early phase of hyperglycemic state, and possibly regulates the NF-κB activity (105). Our previous data shows that db/db mice exhibited a significant reduction of blood flow in peripheral nerve tissues, which were measured using laser Doppler flowmetry. This reduction in blood flow correlated with nerve dysfunction and axonal demyelination in a sex-dependent manner (106). However, the delivery of miR-146a mimics significantly improved peripheral nerve tissue perfusion (63). These data highlight the role of miRNAs in restoring blood flow which eventually improves the nerve function in the treatment of DN (Table 2).

**Table 2:** miRNAs and the neurovascular dysfunctions in DN.

**miRNAs**	**Chromosomal location**	**Target genes/regulated pathway**	**Function**	**References**
Let-7i	chr10 (mouse)	Unknown	Promotes axonal growth of DRG neurons	(75)
miR-29b (miR-29a/b1 cluster)	chr6 (mouse)	TGF-β/Smad3 pathway	Protects DRG neurons against apoptosis; promotes axonal growth of DRG neurons	(77)
miR-29c (miR-29b2/c cluster)	chr1 (mouse)	PRKCI	Promotes axonal growth of DRG neurons	(80)
miR-145-3p (miR-143/145 cluster)	chr18 (rat)	PI3K/AKT/mTOR pathway	Protects SCs against apoptosis	(97)
miR-199a (miR-199a/214 cluster)	chr19 (human)	SerpinE2, MMP-13	Promotes coagulation	(104)
miR-146a	chr11 (mouse)	TRAF6, IRAK1	Reduces inflammatory response	(63)

### miRNAs as Biomarkers in the Diagnosis of Diabetic Neuropathy

The diagnosis of DN is not yet standardized but comprises both qualitative and quantitative methods. In addition to being evaluated for any signs of muscle weakness, numbness, and impaired reflexes, patients are usually administered blood tests (vitamin level, diabetes, and immune function), imaging tests [computed tomography (CT) scans, magnetic resonance imaging (MRI), muscle and nerve ultrasound], non-invasive neurological tests (electromyography and nerve conduction velocity test), and invasive tests (including nerve biopsy and skin biopsy) (107–110).

miRNA profiles of diabetic patients have been reported in various studies. Liu et al. measured miRNA profiles in the mononuclear cells collected from 63 diabetic patients with or without complications. The study confirmed that miR-125a-5p, miR-145-3p, miR-99b-5p, and miR-873-5p were enriched in peripheral blood mononuclear cells, and the targeted gene families of these miRs were associated with DN (22). A comprehensive study on the serum miRNA profile of type 1 diabetes patients with DN revealed that serum miR-518d-3p and miR-618 were upregulated compared to diabetic patients without DN (23). Another study of 60 diabetic patients demonstrated a correlation between miR-199a-3p and reduced extracellular serine content (104). Ciccacci et al. reported that patients carrying the rs3746444 GG genotype of the miR-499a had a higher risk of developing DN (111). In a study of DN patients, the expression of miR-21-5p, miR-146a, and miR-155 were aberrant in white blood cells, peripheral nerves and skin tissue (112) (Table 3).

**Table 3:** miRNAs as biomarkers of diabetic neuropathy.

**miRNAs**	**Origin**	**Expression**	**References**
miR-873-5p, miR-125a-5p, miR-145-3p, miR-99b-5p	Blood mononuclear cells	↑	(22)
miR-518d-3p, miR-618	Serum	↑	(23)
miRNA-199a-3p	Plasma	↑	(104)
miR-499a with rs3746444 SNP	Peripheral blood	↑	(111)
miR-330-5p, miR-17-1-3p, miR-346	DRG	↑	(113)
miR-21, miR-29a/b/c, miR-192	Serum	↑	(114)
miR-146	Blood mononuclear cells, lower leg skin, white blood cells	↓	(112, 115)
miR-203, miR-181a-1, miR-541	Spinal dorsal horn	↓	(116)
miR-341	DRG	↑	(116)
miR-155	Sciatic nerve, blood, white blood cells, lower leg skin	↓	(60, 112)
	Sural nerve	↑	
miR-21	White blood cells, sural nerve	↑	(112)

Some groups have identified dysregulated miRNAs in the tissue of animal models of DN. Guo et al. examined microRNA and mRNA expression profiling in the dorsal root ganglia isolated from diabetic rats and built a microRNA-gene network. They found that miR-17-1-3p, miR-330-5p, and miR-346 are potent promoters of DN (113). Xourgia et al. proposed that miR-146, miR-499a, and miR-199a-3p are possible biomarkers of DN (21). In addition, miR-181a-1, miR-541, miR-341, and miR-203 were reported to be dysregulated in an animal model of DN (116). These data suggest that circulating miRNAs may serve as potential biomarkers in the diagnosis of DN.

## Extracellular miRNAs and Diabetic Neuropathy

### miRNAs and Extracellular Vesicles

Since miRNAs have been detected in biological fluid, their characteristic properties have been investigated in order to identify their potential as biomarkers or therapeutic targets (117). The stability of circulating miRNAs supports its potential as biomarkers. There are two possible means by which miRNAs can be protected from RNase degradation: complexing with Argonaute-protein, and being packaged within extracellular vesicles (EVs) or lipoprotein (118). Exosomes have low immunogenicity, low toxicity, and high nucleic acid loading capacity. Therefore, they hold great promise as vehicles for therapeutic cargos (119). Exosomal miRNAs have been found to regulate target gene expression and thereby mediate physiological responses in the recipient cells (120). Some studies employed silencing of Dicer to generate miRNA-depleted EVs, and the effects of the naïve exosomes were abolished (121, 122). These studies suggest that the miRNA cargo of exosomes largely contribute to their molecular and biological effects. According to www.Clinicaltrail.gov, there are several ongoing clinical trials using exosomal miRNAs to diagnose or to study the pathophysiology of myocardial infarction, diabetic retinopathy, cancer, and sepsis (NCT04127591, NCT03264976, NCT03738319, NCT03911999, NCT02957279).

### Extracellular miRNAs in Peripheral Neurovascular Function

In a preclinical study, Lopez-Verrilli et al. reported that Schwann cell-derived exosomes were internalized by DRG axons to promote axonal regeneration (123). Our group showed that exosomes derived from Schwann cells (SC-exos) increased intraepidermal nerve fiber density in the footpad and reduced axonal and myelin damage of the sciatic nerve, leading to a reduction of neuropathic symptoms in DN mice (124). In contrast, exosomes derived from Schwann cells cultured in high-glucose medium facilitated the development of DN by reducing epidermal nerve fibers (125). Naïve SC-exos were enriched in miR-21, miR-27a, and miR-146a. However, the ablation of miR-27a in exosomes abolished their effects on promoting axonal growth of diabetic DRG neurons and migration of Schwann cells compared with naïve SC-exos. Bioinformatics analysis revealed that miR-21, miR-27a, and miR-146a target Semaphorin 6A (SEMA6A), Ras homolog gene family, member A (RhoA), phosphatase and tensin homolog (PTEN), and nuclear factor-κB (NF-κB) genes, respectively, thereby protecting axons and improving axonal growth. Exosomes isolated from Schwann cells stimulated by high glucose were enriched for miR-28, miR-31a, and miR-130, which target DNA methyltransferase-3a (NDNMT3A), NUMB (endocytic adaptor protein), synaptosome associated protein 25 (SNAP25), and growth-associated protein-43 (GAP43), separately and participate in axonal growth (125).

### Extracellular miRNA as Therapy Target for Diabetic Neuropathy

Mesenchymal stromal cell (MSC)-derived exosomes have been employed in the study of cancer, stroke and cardiovascular diseases. These studies showed that MSC-exosomes function via their miRNA cargoes (126). In a recent study, we administered MSC-derived exosomes to db/db mice with DN and observed a significant improvement in neurological outcomes (127). Further analysis confirmed that MSC-exosomes inhibit the inflammatory response and promote neurovascular remodeling. Data from miRNA sequence and bioinformatics analysis revealed that MSC-exosomes are enriched in miRNAs that are highly involved in inflammation, cell cycle progression, and apoptosis. Let-7a, miR-23a, and miR-125b, among others, synergistically target the TLR4/NF-κB signaling pathway, thereby regulating macrophage polarization (127).

The use of exosomes as therapy represents an innovative and very promising delivery system. It provides an opportunity to address a compelling clinical need. To mimic the property of exosomes, chemically synthesized nanoparticles have been used to package small RNAs in disease treatment (128). In a study on DN, Luo et al. employed imine backbone-based polymer to construct a cationic nanocarrier for miR-146a and demonstrated that nanoparticle–miR-146a-5p alleviated the morphological changes of sciatic nerve in a rat model of DN by regulating the inflammatory response (70).

## Conclusion and Future Perspective

The field of miRNAs as a potential therapy for DN is growing. In this review, we summarized recent findings on miRNAs that are involved in the pathophysiology and treatment of DN. Several challenges remain to enable the translation of miRNA-based therapy to the clinic. The biological function of the majority of miRNAs has yet to be investigated. Previous studies have targeted specific dysregulated miRNAs. However, given that DN is caused by multiple factors, the regulation of a single miRNA may not be sufficient to reverse the impairments. A network of miRNAs and mRNAs will likely provide more comprehensive and accurate characterization of DN. There is a paucity of literature interrogating the mechanisms by which miRNAs regulate the development of DN. Most of the miRNA-related studies were performed in different rodent strains utilizing different methods to induce DN. More work is needed to unlock the potential of miRNA therapy for clinical use.

Recent preclinical studies using exogenous double-stranded miRNAs mimic or single-stranded antisense RNAs (antimiRs) have shown promising results in rodent models of DN (50, 59, 63, 77, 79). However, the use of miRNAs as translational agents or pharmaco-targets in DN patients requires future investigations. The mechanisms underlying these beneficial therapies are being elucidated. Additionally, safety considerations need to be fully explored. Exosomes enhance the stability of miRNA delivery with high transduction efficiency. Largely, exosome-based therapy appears to be safe without adverse effects. Standard quality control needs to be established to ensure the consistency of exosomal products and the content of their miRNA cargo.

## Author Contributions

BF compose the 1st version of the manuscript. ZZ wrote part 1-2. MC wrote part 3-4. XL is in charge of the overall design and revised the manuscript. All authors contributed to the article and approved the submitted version.

## Conflict of Interest

The authors declare that the research was conducted in the absence of any commercial or financial relationships that could be construed as a potential conflict of interest. The handling editor is currently organizing a research topic with one of the authors XL.
